# Development of new 3D human ex vivo models to study sebaceous gland lipid metabolism and modulations

**DOI:** 10.1111/cpr.12524

**Published:** 2018-11-06

**Authors:** Anne‐France de Bengy, Nico Forraz, Louis Danoux, Nicolas Berthelemy, Sébastien Cadau, Olivier Degoul, Valérie Andre, Sabine Pain, Colin McGuckin

**Affiliations:** ^1^ CTIBIOTECH Meyzieu‐Lyon France; ^2^ BASF Beauty Care Solutions Lyon France

**Keywords:** 3D model, sebaceous gland, sebocyte, sebum, skin lipid metabolism, squalene

## Abstract

**Objectives:**

Sebaceous glands maintain skin homeostasis by producing sebum. Low production can induce hair loss and fragile skin. Overproduction provokes seborrhoea and may lead to acne and inflammatory events. To better study sebaceous gland maintenance, sebocyte maturation, lipid production and ageing or inflammatory processes, we developed innovative 3D ex vivo models for human sebaceous glands.

**Materials and Methods:**

Culture conditions and analytical methods optimized on sebocyte monolayers were validated on extracted sebaceous glands and allowed the development of two 3D models: (a) “air‐liquid” interface and (b) human fibronectin‐coated “sandwich” method. Lipid production was assessed with microscopy, fluorometry or flow cytometry analysis after Nile Red staining. Specific lipids (particularly squalene and peroxidized squalene) were measured by Gas or liquid Chromatography and Mass spectrometry.

**Results:**

This study allowed us to select appropriate conditions and design Seb4Gln culture medium inducing sebocyte proliferation and neutral lipid production. The “air‐liquid” model was appropriate to induce sebocyte isolation. The “sandwich” model enabled sebaceous gland maintenance up to 42 days. A treatment with Insulin Growth Factor‐1 allowed validation of the model as we succeeded in mimicking dynamic lipid overproduction.

**Conclusion:**

Functional sebocyte maturation and physiological maintenance were preserved up to 6 weeks in our models. Associated with functional assays, they provide a powerful platform to mimic physiological skin lipid metabolism and to screen for active ingredients modulating sebum production.

## 1 INTRODUCTION

Human skin homeostasis requires sebum production regulation. Sebum is produced by sebaceous glands (SG) located in skin dermis and often associated with a hair follicle forming a pilo‐sebaceous unit.^1^ Various sebum functions such as skin protection against dehydration^2^ or antibacterial and anti‐oxidant properties^3^, ^4^ are well known. In rodents, a role in thermoregulation and resistance against UVB‐induced apoptosis^5^, ^6^, ^7^ has also been shown. However, those effects remain unclear for human skin^8^ and, even if a real correlation between SG morphology and hair loss has also been shown,^9^ sebum effect on human hair follicle integrity remains unclear.

Sebum is the holocrine secretion formed by glandular cell disintegration into follicular duct of pilosebaceous unit.^1^ Human undifferentiated sebocytes located in SG periphery express cytokeratin 7 (K7).^10^ During maturation, they progressively migrate towards SG centre, accumulating lipid droplets and expressing markers such as Melanocortin 5 receptor (MC5R),^11^ Peroxisome proliferator‐activated Receptor gamma (PPARg)^12^ and Mucin‐1 (Muc1), a well‐known marker of late differentiated sebocytes.^13^

Human sebum corresponds to a mixture of complex lipids:glycerides and free fatty acids (FFA) (57%), cholesterol (2%), squalenes (12%) and wax esters (26%).^1^ Its specific composition compared to other animals^1^ may explain differences in function. Sebocyte lipids are secreted onto skin surface and constitute an important component of skin barrier with keratinocyte‐derived lipids.^1^

Deregulated sebum production leads to oily or dry skin but also to common skin inflammatory diseases including acne, atopic dermatitis and psoriasis.^14^

Considering the differences in sebum function and composition between human and other animals, human experimental SG models are essential for better understanding defects in SG and evaluating active ingredient effects in regulating the right composition of sebum. Fundamental research on human sebaceous cell function and control requires human models in vitro. Isolation of human SG and cultured human sebocytes has been shown to preserve important sebocytic characteristics, although they undergo an incomplete terminal differentiation *in vitro*.^15^ Indeed, to date, no existing in vitro 3D model can produce real sebum with the right lipid composition.

In order to better study SG function and particularly its maintenance, sebocyte maturation, lipid production and additionally some ageing or inflammatory processes, this study aimed to develop innovative models for 3D ex vivo culture of human SG by establishing adequate cell culture conditions and validating qualitative and quantitative methods to investigate neutral lipid production.

## 2 MATERIALS AND METHOD

### 2.1 Sample collection

Skin samples were obtained from human donor surgeries following informed consent and applicable ethical guidelines and regulations.

### 2.2 SG extraction and culture

**Figure 1:**
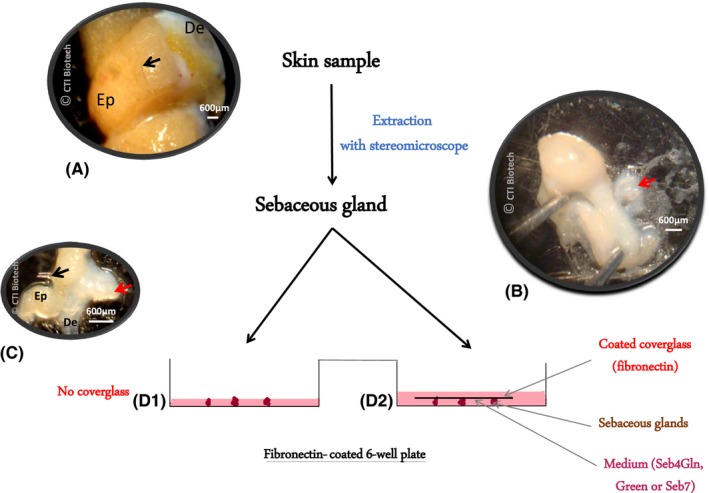
Protocol used for SG isolation and ex vivo culture. A, Skin sample with epidermis (Ep), dermis (De) and a single hair (black arrow). B, C, Progressively isolated SG (red arrow). D, Two SG culture protocols. D1, “Air‐Liquid” 3D model: SG cultivated in a fibronectin‐coated plate with only 600 µL culture medium; D2, “Sandwich” 3D model: SG cultivated between fibronectin‐coated plate bottom and coverglass. Scale bar 600 µm

Sebaceous glands (n = 51) from seven donors (36 months to 59 years old) were microdissected from various skin samples. Epidermal and dermal tissue around SG was progressively removed with scalpels under stereomicroscope in aseptic conditions. SG were then transferred in 6‐well plates, and two methods were used for culture (Figure 1):

*“Air‐liquid”* ex vivo* culture*: SG were cultivated in a fibronectin‐coated plate with 600 µL culture medium so that SG can be at the air–liquid interface (Figure 1D1—modified from *Xia et* *al* ^16^).
*“Sandwich”* ex vivo* culture*: SG were cultivated between fibronectin‐coated plate bottom and fibronectin‐coated coverglass with 1 mL culture medium (Figure 1D2—modified from *Mc Nairn et* *al* ^17^).

In both cases, three glands were grown in each well. Medium was changed every 3‐4 days.

### 2.3 Cell growth

After isolation and amplification, sebocytes were cultured on fibronectin‐coated 6‐well plates (1.3 µg/cm^2^) and grown in different media with sequential subculture as necessary:

*Green*‐based medium^18^ formulated for keratinocyte and epithelial cell culture,
Seb5, a serum‐free medium also used for keratinocyte culture (CTIBiotech, Meyzieu‐Lyon, France),
Seb7 designed for sebocyte proliferation (CTIBiotech),
Seb4Gln (4 µM glutamine) and Seb4 (4 nM glutamine), formulated specifically for both sebocyte proliferation and differentiation (CTIBiotech).

Sebocytes were collected, counted and assessed for viability with LUNA‐FL™ Dual Fluorescence Cell Counter (Logos Biosystems, Villeneuve d'Ascq, France) to establish growth curves. 40 000 cells/well were seeded each time in triplicates for each condition, from passage 2‐5.

### 2.4 Analysis of neutral, total and polar lipid production by sebocytes

#### 2.4.1 Microscopy analysis

After amplification in 96‐well plate, sebocytes were fixed in formaldehyde 4% w/v (Sigma‐Aldrich, Saint Quentin Fallavier, France) and stained with 1 µg/mL 4′,6‐diamidino‐2‐phenylindole (DAPI, Sigma‐Aldrich) and 1 µM Nile Red (Sigma‐Aldrich). Microscopic observations were made with a fluorescence microscope (Eclipse Ti, Nikon, Champigny sur Marne, France).

#### 2.4.2 Fluorometry analysis

**Figure 2:**
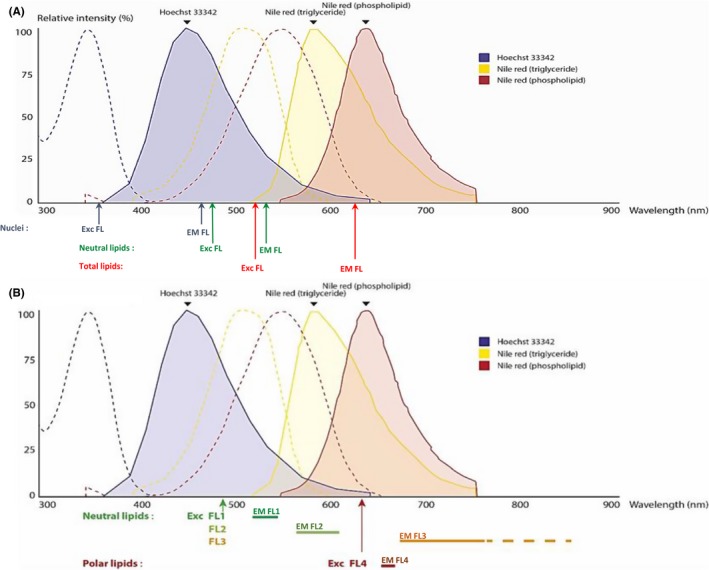
Excitation and emission wavelengths used for lipid analysis compared to fluorescence excitation and emission spectra of Hoechst, Nile Red in triglycerides (neutral lipids) and in phospholipids (polar lipids). Dotted and solid line curves represent, respectively, excitation and emission spectra of Hoechst (blue), Nile Red in triglycerides (yellow) or phospholipids (red). Excitation and emission intensity (relative intensity) are expressed as a percentage of excitation or emission peak (=100%). Under each graph are represented wavelengths used in fluorometry (A) and in flow cytometry (B). Exc FL and EM FL, Excitation and Emission fluorescence wavelengths

After amplification in 96‐well plate, sebocytes were fixed and stained as previously described. Unstained sebocytes were used as control. As Hoechst is known as a nucleus marker and Nile Red as a neutral and/or polar lipid marker, depending on excitation and emission wavelengths,^19^ fluorescence intensity (FI) was assessed with fluorometer (TECAN Infinite M1000, Lifesciences, Männedorf, Switzerland) on replicates (5‐8) with the following wavelength excitation and emission (Figure 2A):

Nuclei (Hoechst): excitation 356 nm—emission 465 nm
Neutral lipids (Nile Red): excitation 475 nm—emission 530 nm
Total lipids (Nile Red) excitation 520 nm—emission 625 nm

#### 2.4.3 Flow cytometry analysis

After amplification in 6‐well plates, sebocytes were trypsinized and stained with Nile Red solution (1 µM). FSC, SSC and Nile Red Intensity Fluorescence Mean (MFI) were analysed with different channels of a flow cytometer (FACSCalibur, BD Biosciences, Le Pont de Claix, France) corresponding to different excitation and emission fluorescence spectra revealing either neutral or polar lipid amount per cell (Figure 2B). Unstained sebocytes were used as control. Each condition was analysed once weekly for 3 weeks.

#### 2.4.4 Statistical analysis

Values represent the mean ± standard deviation. Statistical significance of the data was assessed by Kruskall‐Wallis test. Mean differences were considered significant when *P* < 0.05.

### 2.5 Isolated sebocyte and SG characterization

Cells and SG were first fixed with formaldehyde 4% w/v (Sigma‐Aldrich). Then, they were stained with 4′,6‐diamidino‐2‐phenylindole (DAPI—1 µg/mL), K7 (Novus), Keratin 5 (K5—Novus Biological, Bio‐techne, Lille, France), MC5R (Abcam, Paris, France) and/or Muc‐1 antibodies (Acris Antibody, Herford, Germany). Staining was analysed with either fluorescence microscope (Eclipse Ti—Nikon) or confocal microscope.

### 2.6 Lipid analysis and quantification in the “sandwich” 3D ex vivo SG model

Lipids from SG of two donors were analysed after 6 days in Seb4Gln or *Green* medium. As control, IGF‐1 (100 ng/mL for 6 days) was added in some SG culture medium to induce lipid overproduction. Each experiment was performed with one replicate.

#### 2.6.1 Lipid extraction from SG

Sebaceous glands were grinded in 1 mL methanol, allowing sebaceous lipid extraction, and lipids of methanol solutions were extracted by liquid–liquid exchange with hexane. Then, hexane solvent was evaporated under nitrogen flux and lipid residues were frozen before chemical analysis.

#### 2.6.2 Chemical analysis of sebaceous lipids

Lipid extracts were purified by several extractions with a binary mixture of methanol and chloroform, followed by several washes with a NaCl‐saturated aqueous solution.

Then, neutral lipid analysis (squalene, cholesterol, free fatty acids, waxes, and glycerides) was performed by gas chromatography (Agilent, Les Ulis, France) combined with mass spectrometry (Agilent 5973 N) whereas per‐oxidized squalene were detected by liquid chromatography (Dionex, Thermo Fisher, Dardilly, France) coupled with mass spectrometry (MSQ+ Thermo, Fisher, Dardilly, France).

## 3 RESULTS

### 3.1 Seb4Gln medium stimulates sebocyte growth and neutral lipid production

As sebum production results both from sebocyte proliferation and differentiation, optimized conditions to amplify sebocytes and to induce neutral lipid production (the main components of released sebum) were assessed.

Sebocytes were grown in different conditions and subcultured weekly over 3 weeks from P2 to P5. Among all the media tested, one was formulated specifically for sebocyte culture with a low (Seb4) or a high amount of glutamine (Seb4Gln). Seb5 and Green medium, usually used for keratinocyte culture, were tested as control, as well as Seb7, a sebocyte proliferative medium.

**Figure 3:**
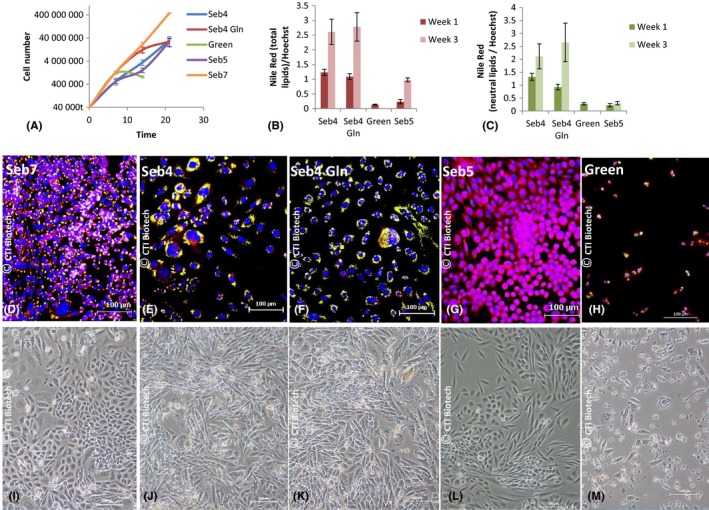
Effect of different media on sebocyte starting P2 and lipid production. Seb7, *Green *and Seb5 media were tested as control. A, Sebocyte growth curves. Average of three biological replicates. Error bars represent sd B‐ Ratio (relative fluorescence) between Nile Red (Excitation 475 nm—Emission 530 nm) and Hoechst Signal (Excitation 356 nm—Emission 465 nm) obtained by fluorimetry and revealing total lipid production. C, Ratio between Nile Red (Excitation 520 nm—Emission 625 nm) and Hoechst Signal revealing neutral lipid production. D‐M, Pictures of sebocytes in brightfield (I‐M) or stained with DAPI and Nile Red (D‐H) after 5 (P5) (D‐G) or four subcultures (P4) (H‐M) in Seb7 (D, I), Seb4 (E, J), Seb4Gln (F, K), Seb5 (G, L) or Green (H, M) medium. In red, total lipid. In yellow, merge of total (red) and neutral lipid (green). In blue, nuclei (DAPI). Scale bar 100 µm

Growth curves (Figure 3A) analysis confirmed that Seb7 induced high sebocyte proliferation and showed that Seb4 could boost sebocyte growth for up to 21 days. Glutamine supplementation increased significantly growth rate: in 14 days sebocytes expanded ~310‐fold in Seb4Gln vs ~88‐fold in Seb4 (*P* = 0.049).

Simultaneously, sebocytes grown in the same conditions were stained weekly with Hoechst or DAPI and Nile Red to assess their differentiation level. Microscopic observations (Figure 3D‐G) strongly suggested that sebocytes produced more neutral lipids when grown in Seb4 or Seb4Gln compared to Seb5 medium. We then developed a fluorometric quantification method where the ratio between Nile Red and Hoechst signal provides an estimation of lipid production amount per cell (Figure 3B,C). As Nile Red excitation and emission spectra depend on lipid type, we used different wavelengths to reveal neutral lipids (exc. 475 nm em. 530 nm) and total lipids (exc. 520 nm em. 625 nm). Figure 3B suggests that total lipid production per cell was higher in Seb4 and Seb4Gln than in Green and Seb5 media and increased overtime in all cases, except for Green medium due to early cell death (around 7 days). Results were similar for neutral lipids (Figure 3C) except that neutral lipid production remained low in Seb5 medium during all the experiment. All together, these data suggest that Seb4 and Seb4Gln stimulated mainly neutral lipid production, whereas Seb5 stimulated polar lipid production.

**Figure 4:**
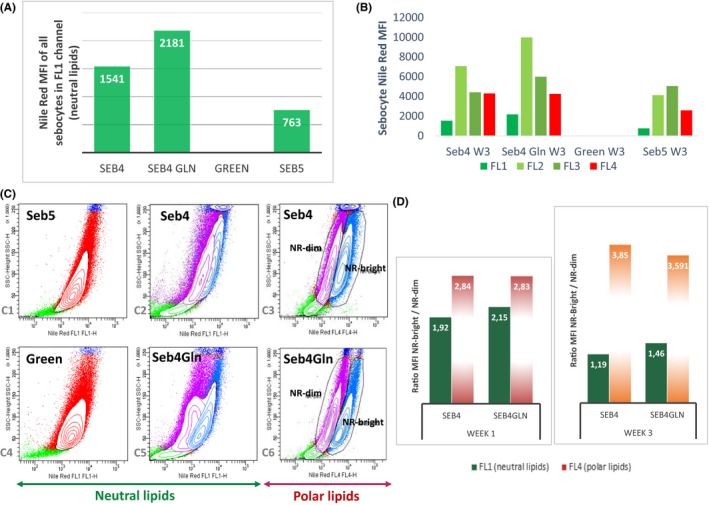
Lipid production by sebocytes and glutamine effect revealed by flow cytometry. A, Neutral lipid production by sebocytes grown 3 weeks with (Seb4Gln) or without glutamine (Seb4) revealed by FL1 channel after Nile Red staining. *Green* and Seb5 media were tested as control. B, Nile Red MFI obtained with the four different channels FL1, FL2, FL3 and FL4 in the same conditions. C, Dot plots showing Nile Red signal in FL1 channel (neutral lipids—C1, C2, C4, C5) or FL4 channel (polar lipids—C3, C6) for sebocytes grown 7 days with (Seb4Gln—C5, C6) or without glutamine (Seb4—C2, C3). Sebocytes grown in Seb5 (C1) and *Green *medium (C4) were used as control. NR‐dim and NR‐bright, sebocyte population with, respectively, low and high Nile Red signal. D, Nile Red MFI ratio between NR‐bright and NR‐dim populations after 7 days (Week1) or 21 days (Week3) of sebocyte culture in Seb4 and Seb4Gln media. One repetition was done each week

In order to confirm those results and estimate more precisely neutral, total and polar lipid production per cell, sebocytes were analysed by flow cytometry. Different channels were used (Figure 2B): FL1 and FL4 to quantify, respectively, sebocytes with neutral and polar lipids, FL2 and FL3 to quantify sebocytes with total lipids including a higher proportion of neutral (FL2) or polar lipids (FL3). Neutral lipid amount per sebocyte appeared 2‐fold higher in Seb4 and 3‐fold higher in Seb4Gln compared to Seb5 medium after 3 weeks of cultivation (Figure 4A). After only 2 weeks, the difference was even more than 5‐fold higher between Seb5 and the other media (data not shown). Moreover, the higher MFI was obtained with FL2 channel in Seb4 and Seb4Gln media after 2 (data not shown) and 3 weeks of cultivation (Figure 4 B). This reinforces the idea that sebocyte lipid production depends on the medium used, with a higher proportion of neutral lipids in Seb4 and Seb4Gln media.

Furthermore, although only one population was detected in FL 1 channel (Figure 4C1,C4) and in FL4 channel (data not shown) in Green or Seb5 medium, flow cytometry revealed heterogeneity of lipid production in sebocytes grown one week in Seb4 or Seb4Gln (Figure 4C). In Seb4Gln, two distinct populations expressing low (NR‐dim) or high Nile Red signal (NR‐Bright) were observed in FL4 (Figure 4C6) and FL1 channels (Figure 4C5). NR‐bright population identified a more differentiated sebocyte population producing more neutral and polar lipids when NR‐dim population was composed of less differentiated and probably actively proliferating sebocytes. In Seb4, these two populations were clearly visible with FL4 channel (Figure 4C3) but not so clearly with FL1 channel (Figure 4C2) suggesting a positive effect of glutamine on neutral lipid production.

By calculating the ratio between NR‐bright and NR‐dim population MFI (mean of fluorescence intensity) after 1 or 3 weeks of culture in both media (Figure 4D), we showed that this ratio was always slightly higher in Seb4Gln medium for neutral lipids (FL1). This confirms the positive effect of glutamine on neutral lipid production.

### 3.2 The “air‐liquid” 3D ex vivo SG model was suitable for sebocyte isolation

**Figure 5:**
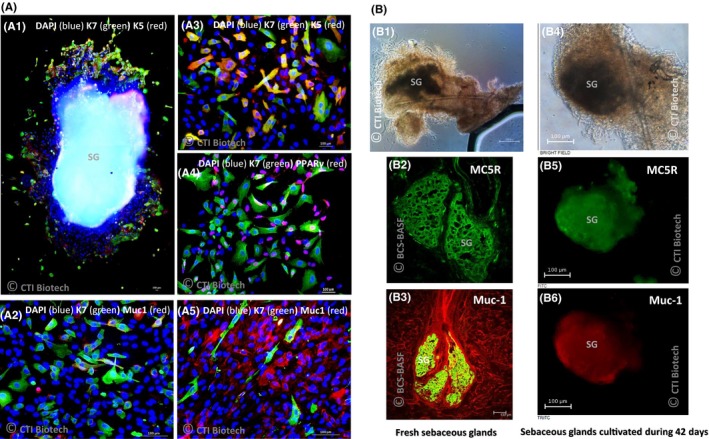
Staining of isolated sebocytes obtained with “air‐liquid” method (A) and SG cultivated during a long period with “sandwich” method (B). A, Isolated sebocytes growing in Seb4Gln, around a SG from an abdomen skin sample (A1), after 3 (A2‐A4) or 5 subcultures (A5). B, *Fresh* (B1‐B3) and ex vivo cultivated SG during 42 days with “sandwich” method (B4‐B6) observed before (B1) or after staining (B2‐B6) with MC5R (B2, B5—green) or Muc‐1 antibody (B3—Green, B6—red). B2: Brightfield control. Photos obtained with confocal microscope (B2, B3) or fluorescence microscope (A1‐A5, B1, B4‐B6). DAPI: 4′,6‐diamidino‐2‐phénylindole, K7: cytokeratin 7, K5: cytokeratin 5, Muc 1: Mucin‐1, MC5R: Melanocortin 5 Receptor, PPARγ: Peroxisome proliferator‐activated Receptor gamma. Scale bar 100 µm

The “air‐liquid” 3D ex vivo SG model developed in this study enabled isolation of low differentiated sebocytes expressing K7 (Figure 5A1). Moreover, K7 expression is maintained for some cells during successive subcultures (Figure 5A2) and only K7‐positive cells express K5 (Figure 5A1,A3). As K5 is also expressed by keratinocytes but not K7, it highlights the absence of keratinocyte contamination. A positive staining of Muc‐1 depending on sebocyte differentiation state (Figure 5A2,A5) and PPARg (Figure 5A4) was also detected. Such cells were isolated only in Seb4Gln medium. For sebocyte isolation, this method gave better results than “sandwich” method. Moreover, some cells also expressed K7 in the sebaceous gland after 33 days of culture, demonstrating successful maintenance of SG integrity.

On the contrary, the “sandwich” 3D ex vivo SG model did not enable sebocyte isolation. But it allowed for long‐term maintenance of SG as no alteration of their structure was noticed by microscopic observations (Figure 5B4) after 42 days when compared to freshly isolated glands (Figure 5B1). Compared to fresh (in situ) SG immunostained as control (Figure 5B2,B3), 3D ex vivo SG cultivated in Seb4Gln medium (Figure 5B5,B6) preserved the expression of maturation marker MC5R and Muc‐1.

### 3.3 Seb4Gln medium induced sebum lipid production and particularly squalene in 3D ex vivo SG models

**Figure 6:**
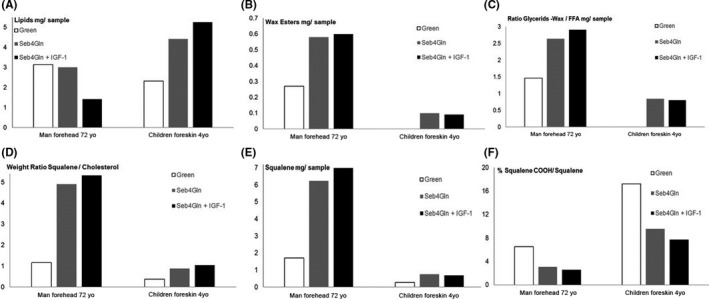
Lipid quantification in SG after 6 days’ maintenance according to skin origin and culture media (*Green* or Seb4Gln media supplemented or not with IGF‐1)

SG lipid composition of a 4‐year‐old child (foreskin) and a 72‐year‐old adult (abdomen) was studied in *Green* or Seb4Gln medium supplemented or not with IGF‐1 (100 ng/mL). Figure 6 shows that adult SG contained less total lipids (Figure 6A) but more sebaceous specific lipids, (Wax esters, Glycerides‐Wax/FFA and squalene—Figure 6B‐E) than child’s SG in all three‐tested media. Nevertheless, for both samples, SG in Seb4Gln contained more specific lipids than in *Green* medium (Figure 6B‐E). Moreover, peroxidized squalene/squalene ratio (Figure 6F) demonstrated that seb4Gln medium favoured squalene production and delayed peroxidized squalene formation compared to Green medium. We further showed that IGF‐1 moderately induced production of the different kinds of lipids in adult sample.

## 4 DISCUSSION

The objective of this study was to develop reliable methods to study human sebocyte metabolism and lipid production in 3D ex vivo SG models. Such models are highly sought after in dermatological, active ingredients and pharmaceutical research laboratories.

Culture conditions and analytical methods were first optimized using 2D human sebocyte monolayers, enabling us to develop specific “in house” proprietary medium supporting both cell proliferation and production of neutral lipids that are specific of SG. We further showed that the defined medium was suitable for our 3D models to maintain SG morphology and integrity in culture.

In this first part, comparison of different culture media in 2D and 3D ex vivo cell systems suggested that high glutamine supplementation had positive effects on sebocyte growth and neutral lipid production. Modulating glutamine content in sebocyte culture conditions provides a high‐definition switch to artificially stimulate (high glutamine content Seb4Gln medium) or to slow down (low glutamine content Seb4 medium) sebum’s neutral lipid production.

Glutamine has long been known to have an effect on mammalian cell growth.^20^, ^21^ Glutamine had also been reported to be involved in lipid metabolism but for cells in hypoxic conditions^22^ which were not the case in this study. Glutamine appeared to have a dual effect by initially stimulating sebocyte proliferation (week 1) and then stimulating neutral lipid production (week 3) whilst diminishing the proliferative effect.

This was confirmed by all analytical methods developed and used in this study with Nile Red/Hoechst staining: qualitative microscopy, semi‐quantitative fluorometry and novel flow cytometric analysis. Fluorometry enables more precise semi‐quantitation of neutral and total lipid content in different conditions than fluorescence microscopy. However, flow cytometric analysis of neutral and total lipid content described herein provided an even more powerful tool to finely evaluate sebocyte proliferation versus differentiation in 2D or 3D cell systems. Nile Red has large excitation and emission spectra. Low wavelength mainly reveals neutral lipids analysed at each single cell level. Nile Red Mean Fluorescence Intensity (MFI) analysis demonstrated that high glutamine Seb4Gln medium maintained a more differentiated sebocyte population producing more neutral lipids (NR‐bright cells compared to NR‐dim less differentiated and probably actively proliferating sebocytes).

In the second part of the study, we demonstrated that the “Air‐Liquid” 3D ex vivo SG model cultivated in fibronectin‐coated plate to anchor the SG was the most adapted to induce sebocyte isolation and amplification. The proliferation reached up to 33 days with maintenance of the gland explant’s morphology and integrity using Seb4Gln medium. After subculture isolated sebocytes grew fast in the same medium, still expressing K7 human sebocyte immature marker^4^, ^10^ for a period and then progressively expressing human sebocyte mature markers such as PPARg^12^, ^17^ or Muc‐1.^4^, ^13^

The “sandwich” ex vivo SG culture between fibronectin‐coated plate and coverglass‐enabled long‐term maintenance of SG for up to 42 days with sebocytes expressing Muc‐1 and MC5R. Ex vivo culture of SG was previously done by various teams in order to isolate primary sebocytes^16^, ^17^, ^23^, ^24^ which were then used to better understand in vivo sebum production and modulation. The “sandwich” method presented here provides a more suitable model to mimic the physiological maturation of the SG to study its metabolism. This long‐term 3D human ex vivo SG model was further successfully evaluated using various functional assays: Nile Red labelling (microscopy, fluorimetry, flow cytometry) for total lipid content and neutral lipid identification, including squalene and peroxidized squalene quantified, respectively, by GC/MS and LC/MS Lipid analysis of two skin samples revealed a higher amount of sebaceous specific lipids and lower amount of oxidized squalene revealing a higher sebum quality in adult donor. Child’s SG probably include more polar/membrane lipids (sebaceous non‐specific lipids). New experiments are needed to determine the cause but it may depend on donor, skin area or more probably on a sebocyte age‐related differentiation. Considering low squalene quantity, higher oxidized squalene percentage may also be an artefact. Nevertheless, for both skin samples, Seb4Gln medium stimulated squalene and wax ester production, showing its interest to allow right sebum production. Squalene peroxidation plays a key role in various skin diseases such as photoinduced skin damage,^25^ acne^26^ Dandruff.^27^ We also demonstrated that Seb4Gln medium limited squalene peroxidation compared to *Green* medium regardless of age and location. Therefore, Seb4Gln may favour anti‐oxidant components preserving sebum for peroxidation. Moreover, treatment with IGF‐1 validated the models as we succeeded in mimicking dynamic lipid overproduction.

Previous models with primary^16^, ^17^, ^28^, ^29^ or immortalized sebocytes^30^, ^31^, ^32^, ^33^ have already been proposed and immortalized sebocytes were successfully used in 3D reconstructed models to improve epidermis structural integrity^34^ and obtain differentiated sebocytes producing lipids or organoids^35^ useful to better understand SG function. However, to our knowledge, no 3D reconstructed model has been proven to produce a sebum with the right composition.

This study show that our 3D ex vivo SG models allow for achieving and preserving functional sebocyte maturation. Moreover, they are suitable for longer term evaluation than other models as physiological maintenance is preserved up to 6 weeks (vs around 2 weeks for ex vivo skin biopsies or immortalized sebocytes in 3D^33^, ^34^, ^35^).

These novel 3D human ex vivo SG models, and associated functional assays, provide a powerful platform to mimic physiological skin lipid metabolism and to screen for and assess the efficacy of modulating active ingredients to balance excess or lack of sebum in conditions closer to in vivo conditions (more predictive and representative efficacy results).

These methods could be further used to study 2D sebocytes monolayers or 3D ex vivo SG models’ behaviour when exposed to stress, cell culture conditions, ingredients or finished products.

## CONFLICT OF INTEREST

The authors declare they have no conflict of interest related to this study.
